# Magnetic Resonance Neurography Reveals Smoking-Associated Decrease in Sciatic Nerve Structural Integrity in Type 2 Diabetes

**DOI:** 10.3389/fnins.2021.811085

**Published:** 2022-02-15

**Authors:** Johann M. E. Jende, Christoph Mooshage, Zoltan Kender, Stefan Kopf, Jan B. Groener, Sabine Heiland, Alexander Juerchott, Peter Nawroth, Martin Bendszus, Felix T. Kurz

**Affiliations:** ^1^Department of Neuroradiology, Heidelberg University Hospital, Heidelberg, Germany; ^2^Department of Endocrinology, Diabetology and Clinical Chemistry, Heidelberg University Hospital, Heidelberg, Germany; ^3^German Center of Diabetes Research, München-Neuherberg, Germany; ^4^Division of Experimental Radiology, Department of Neuroradiology, Heidelberg, Germany; ^5^Joint Institute for Diabetes and Cancer at Helmholtz-Zentrum Munich and Heidelberg University, Heidelberg, Germany; ^6^Department of Radiology (E010), German Cancer Research Center, Heidelberg, Germany

**Keywords:** smoking, diabetic polyneuropathy, magnetic resonance neurography (MRN), type 2 diabetes, diffusion tensor imaging

## Abstract

**Objective:**

It is controversially discussed in how far smoking contributes to diabetic polyneuropathy (DPN) in type 2 diabetes (T2D). Diffusion-weighted magnetic resonance neurography (MRN) at 3 Tesla has been shown to provide objective values for structural nerve integrity in patients with T2D. The aim of this study was to investigate the contribution of cigarette smoking on structural nerve integrity in T2D.

**Methods:**

This cross-sectional prospective cohort study investigated the structural integrity of the sciatic nerve in 10 smokers, 40 never-smokers, and 20 ex-smokers with T2D and 10 healthy control subjects, using diffusion tensor imaging MRN at 3 Tesla and semi-automated nerve fiber tracking. Results were correlated with clinical, electrophysiological, and serological data.

**Results:**

The sciatic nerve’s fractional anisotropy (FA), a parameter for structural nerve integrity, was significantly lower in smokers with T2D when compared to controls (*p* = 0.002) and never-smokers (*p* = 0.015), and lower in ex-smokers when compared to controls (*p* = 0.015). In addition, sciatic nerve radial diffusivity, a marker of myelin damage, was increased in smokers versus controls and never-smokers (*p* = 0.048, *p* = 0.049, respectively). Furthermore, FA in T2D patients was negatively correlated with clinical and electrophysiological markers of DPN. FA also showed negative correlations with the pulse wave velocity, a marker of arterial stiffness and associated microangiopathy, in controls (*r* = −0.70; *p* = 0.037), never-smokers (*r* = −0.45; *p* = 0.004), ex-smokers (*r* = −0.55; *p* = 0.009), and a similar trend in smokers (*r* = −0.63; *p* = 0.076). Negative correlations were found between FA and skin auto-fluorescence, a marker of tissue advanced glycation end product accumulation and therefore long-term glycemic stress in T2D, in never-smokers (*r* = −0.39; *p* = 0.020) and smokers (*r* = −0.84; *p* = 0.004), but not in ex-smokers (*r* = −0.07; *p* = 0.765).

**Conclusion:**

The findings indicate that smoking contributes to sciatic nerve damage in T2D, potentially worsening DPN due to glycemic stress and less microangiopathy-associated myelin damage in active smokers, while angiopathic effects predominate in ex-smokers. To stop smoking may therefore pose a promising preventive measure to slow the progression of DPN in T2D.

## Introduction

Distal symmetric diabetic polyneuropathy (DPN) is one of the most disabling complications of diabetes with increasing prevalence affecting about 200 million patients worldwide causing high morbidity and extensive healthcare costs (Alleman et al., 2015). The poor effect of adjusting serum glucose on the course of DPN in type 2 diabetes (T2D) indicates that pathophysiological mechanisms other than the effects of hyperglycemia play an important role in the development of DPN (Tesfaye et al., 2005; Toth et al., 2012). Although several additional clinical and serological risk factors for developing DPN such as obesity, hypertension, hyperglycemia, dyslipidemia, and a decrease in renal function have been identified in clinical studies, the exact pathophysiological mechanisms underlying DPN remain poorly understood. As a result, there still is a lack of sufficient therapeutic strategies and preventive measures for this disorder (Gaede et al., 1999; Tesfaye et al., 2005; Elliott et al., 2009).

A broadly accepted hypothesis assumes that, in addition to glycation of surface molecules of axons and Schwann cells induced by hyperglycemia, nerve ischemia as a consequence of microangiopathy is another major contributor to nerve damage and the occurrence of painful symptoms in DPN, especially in T2D (Shillo et al., 2019). In this context, it has been discussed controversially whether cigarette smoking as a potential cause for microangiopathy contributes to damage of peripheral nerves in DPN (Benbow et al., 1997; Clair et al., 2015). Although several clinical studies concluded that smoking increases the risk for DPN, and it was found recently that smoking is associated with structural changes in dorsal root ganglia (Jende et al., 2020c), it has not yet been possible to quantify the amount and extent of structural nerve damage caused by smoking *in vivo*. Also, it remains to be determined whether the main contributing factor to nerve damage caused by smoking in T2D is either microangiopathy as a consequence of oxidative stress to the endothelium or oscillations of blood glucose induced by smoking (Csordas and Bernhard, 2013; Christensen et al., 2020).

It is further unknown whether damage to peripheral nerves caused by smoking is reversible once a patient stops smoking (Clair et al., 2015). Recent studies on high resolution magnetic resonance neurography (MRN) at three Tesla (3T) found that, in spite of the progression of clinical symptoms from distal to proximally, the maximum of visible nerve lesions occurs in the sciatic nerve at thigh level (Jende et al., 2020b). It has further been demonstrated that parameters obtained from diffusion tensor imaging (DTI) can serve as reliable parameters for the assessment of structural nerve integrity in T2D (Heckel et al., 2015). In particular, previous studies found that the DTI fractional anisotropy (FA) of peripheral nerves was positively correlated with electrophysiological parameters of both axonal and myelin sheath integrity, whereas axial diffusivity showed positive correlations with parameters of axonal integrity and radial diffusivity showed negative correlations with parameters of myelin sheath integrity (Jende et al., 2020a).

The aim of this study was to investigate the impact of smoking on the sciatic nerve’s structural integrity in patients with T2D by combining diffusion-weighted 3T MRN of the sciatic nerve with detailed clinical, serological and electrophysiological parameters obtained from smokers, ex-smokers and never-smokers with T2D as well as healthy controls.

## Materials and Methods

### Study Design and Participants

This study was approved by the local ethics committee (clinicaltrials.gov identifier NCT03022721) and all participants gave written informed consent. Participants were screened and recruited at the outpatient clinic of the Department of Endocrinology at Heidelberg University Hospital, where all clinical, serological, and electrophysiological examinations took place. Thereafter, patients underwent MRN at the Department of Neuroradiology at Heidelberg University Hospital, where image processing was performed. All patient data were pseudonymized and participating researchers at the Department of Neuroradiology were completely blinded to all patient data. In total, 70 patients with T2D (10 active smokers, 20 ex-smokers, 40 never-smokers; female: 31; male: 39) and 10 age-matched controls (7 women, 3 men) took part in this single-center study between September 2016 and June 2020. Patients with T2D were divided into three groups (smokers, ex-smokers, and never-smokers). Smokers were identified first, and, thereafter, never-smokers and ex-smokers were matched for age, sex, body-mass index (BMI), hemoglobin A1c (HbA1c) levels, cholesterol levels, parameters of renal function, medication, and disease duration, in order to minimize confounding factors that might influence structural nerve integrity. Matching was accepted as adequate when no significant difference was found for each pair-wise group comparison. Recruitment proved to be time-consuming due to difficulties in enrolling smokers with T2D to undergo the extensive study protocol, the subsequent detailed matching process of ex-smokers, never-smokers, and healthy participants, as well as the COVID-19 pandemic.

**FIGURE 1:**
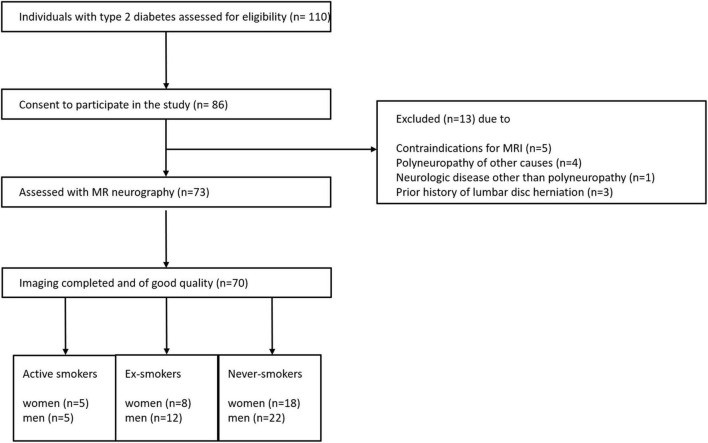
Patient recruitment flowchart.

The control group was matched to the three groups for age, BMI, lipid profile, and parameters of renal function. Detailed information on patient recruitment is given in Figure 1.

For all participants, exclusion criteria were age <18, pregnancy, any contraindications for MR imaging, any history of lumbar surgery or disk protrusion, any other risk factors for neuropathy such as alcoholism, malignant or infectious diseases, hypovitaminosis, monoclonal gammopathy, any previous or ongoing exposure to neurotoxic agents, and any chronic neurological diseases such as Parkinson’s disease, restless leg syndrome, or multiple sclerosis. To exclude severe renal insufficiency as potential confounders, only patients with an estimated glomerular filtration rate (eGFR) > 60 ml/min were included in this study.

### Clinical and Electrophysiological Examination

For every patient, a detailed medical history was documented. Patients with T2D who had never smoked were assigned to the group of never-smokers, patients who had stopped smoking >1 year ago were assigned to the group of ex-smokers and patients who were actively smoking were assigned to the group of smokers. Control subjects who had never smoked were assigned to the control group.

An examination of neuropathic symptoms was performed according to the guidelines issued by the German Society for Diabetology, including evaluation of the neuropathy disability score (NDS) and the neuropathy symptom score (NSS) (Young et al., 1993).

The electrophysiological examination (Viasys Healthcare VikingQuest^®^, Viasys Healthcare GmbH, Höchberg) of the right leg included: distal motor latencies (DML) of the right tibial and peroneal nerve, motor and sensory amplitudes [compound muscle action potentials (CMAPs), and sensory nerve action potentials (SNAPs), respectively] of the tibial, peroneal, and sural nerves, and nerve conduction velocities (NCVs) of the tibial, peroneal, and sural nerves. All examinations were performed in accordance with international standards for electrophysiological examinations and with specifications as issued by the manufacturer of the testing device, see also Stålberg et al. (2019) and Natus Medical Incorporated (2020) for further details. It was assured that skin temperature was at least 32°C throughout the examination.

The intima media thickness (IMT) was assessed with duplex ultrasonographic examination of both carotid arteries (SonoAce X8; Samsung Group). The pulse wave velocity was calculated using non-invasive blood pressure measurements of the arms and ankles (ABI System 1000; Boso d.o.o.).

Dermal accumulation of advanced glycation end products (AGE) was assessed non-invasively measuring skin auto fluorescence (SAF) with an AGE reader (AGE Reader SU, DiagnOptics BV, Netherlands). A skin surface of about 4 cm^2^ was assessed at the volar surface of the forearm (Fernando et al., 2019). For the calculation of SAF, the average emitted light intensity per nm was divided by the average extinction light intensity per nm and multiplied by 100. SAF is expressed in arbitrary units (AU) (Corine van de Zande et al., 2020). Blood was drawn in fasting state and processed immediately under standardized conditions in the central laboratory of Heidelberg University Hospital. Albumin excretion in urine was detected in morning spot urine within all participants. Estimated glomerular filtration rate was calculated with the CKD-EPI-formula (Levey et al., 2009).

### Magnetic Resonance Neurography Imaging Protocol

All participants underwent high-resolution MRN of the right leg in a 3.0 Tesla MR-scanner (Magnetom TIM-TRIO, Siemens, Erlangen, Germany). A 15-channel transmit-receive extremity coil was used, and the following sequence protocol was applied:

(1) an axial high resolution T2-weighted turbo spin echo 2D sequence with spectral fat saturation of the right mid-thigh and the following parameters: repetition time (TR) 5970 ms, echo time (TE) 55 ms, field of view (FOV) 160 × 160 mm^2^, matrix size 512 × 512, slice thickness 4 mm, interslice gap = 0.35 mm, voxel size 0.3 × 0.3 × 4.0 mm^3^, 3 averages, 24 images.

(2) DTI with an axial fat-suppressed, diffusion-weighted two-dimensional echo-planar sequence with the following parameters: TR = 5100 ms; TE = 92.8 ms; b = 0 and 1000 s/mm^2^; directions = 20; FOV = 160 × 160 mm^2^; matrix size = 128 × 128; slice thickness = 4 mm; voxel size = 1.3 × 1.3 × 4 mm^3^; no interslice gap, 3 averages, 24 slices, 1512 images.

### Image Post-processing

**FIGURE 2:**
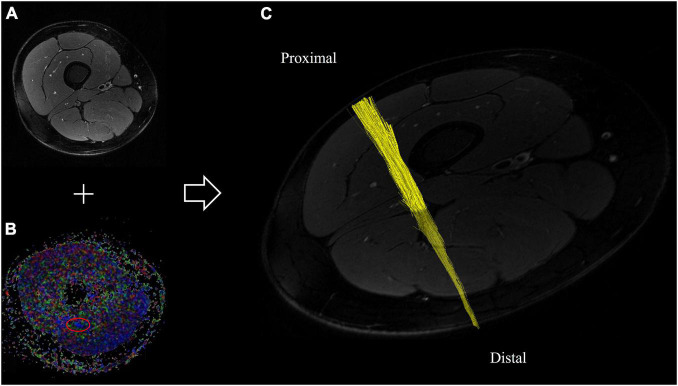
Automated segmentation of the sciatic nerve based on diffusion-weighted and T2-weighted imaging. **(A)** T2-weighted cross-sectional image of the right thigh, and **(B)** corresponding fractional anisotropy map. The sciatic nerve is delineated in red. **(C)** Reconstructed fiber tracts of the sciatic nerve based on diffusion-tensor imaging of the proximal thigh.

All images were pseudonymized and subsequently analyzed in an automated approach using Nordic BRAINEX (NordicNeuroLab AS, 2019), a United States Food and Drug Administration–approved processing software designed for automated calculation and reconstruction of fiber tracts in diffusion-weighted imaging (Christidi et al., 2016). A total of 84 × 1536 = 129,024 images were analyzed accordingly. T2-weighted and diffusion-weighted sequences were co-registered, and the region of the sciatic nerve was marked by two trained neuroradiologists with 6 and 2 years of experience in MRN imaging, respectively. The nerve was automatically segmented with a threshold of >0.1 for the nerve’s FA, a dimensionless quantity that measures directed diffusion, with values between 0 (isotropic diffusion) and 1 (diffusion in only one direction). A tract turning angle of 41.4 degrees, a minimum fiber length of 20 mm, and one seed per voxel were chosen, as done in previous studies (Jende et al., 2021). After nerve fibers were tracked, the nerve tensor eigenvalues λ1, λ2, and λ3 and the average FA were automatically determined by Nordic BRAINEX. Axial diffusivity (AD), radial diffusivity (RD), and mean diffusivity (MD) were subsequently calculated based on the obtained tensor eigenvalues as AD = λ1, RD = (λ2 + λ3)/2, and MD = (λ1 + λ2 + λ3)/3. An illustration of the process of automated nerve segmentation is given in Figure 2.

### Statistical Analysis

Statistical data analysis was performed with GraphPad Prism 6. All data were tested for Gaussian normal distribution using the D’Agostino-Pearson omnibus normality test. If a Gaussian normal distribution was given, *t*-tests were used for comparisons of two groups, one-way ANOVAs with Tukey correction for multiple comparisons were applied for comparisons of more than two groups and Bonferroni-corrected Pearson correlation coefficients were calculated for correlation analysis. If data were not Gaussian distributed, the Mann–Whitney test was used for comparisons of two groups, the Kruskal–Wallis test with Dunn correction for multiple comparisons was used for multiple comparisons of more than three groups and non-parametric Spearman correlations were applied for correlation analysis. For all tests, the level of significance was defined at *p* < 0.05.

## Results

### Clinical and Epidemiological Data

Ten active smokers (5 women, 5 men, 15.79 pack years ± 14.19; range: 1–57, mean age 53.20 ± 12.85 years, BMI 30.48 ± 3.60 kg/m^2^, disease duration 10.60 ± 12.30 years) with T2D, 20 ex-smokers (8 women, 12 men; 20.09 ± 12.05 years after smoking, mean age 59.42 ± 6.87 years, BMI 28.42 ± 5.52 kg/m^2^, disease duration 11.79 ± 10.54 years) with T2D, 40 never-smokers (18 women, 22 men, mean age 57.44 ± 14.01 years, BMI 28.45 ± 5.02 kg/m^2^, disease duration 12.93 ± 11.21 years) with T2D, and 10 age matched controls (7 women, 3 men, mean age 49.80 ± 12.89 years, BMI 28.05 ± 5.69 kg/m^2^) took part in this study. For all groups, ANOVA showed no differences for age (*p* = 0.186), sex (*p* = 0.151), BMI (*p* = 0.678) or NDS scores (*p* = 0.103).

For T2D patient groups, no significant difference in disease duration was found (*p* = 0.744). Oral antidiabetic medication (metformin) was used by 4 smokers, 8 ex-smokers, and 13 never-smokers. Insulin was used by 4 smokers, 5 ex-smokers, and 11 never smokers. A total of 42 participants with T2D suffered from hypertension. Anti-hypertensive medication (beta blockers, renin-angiotensin-aldosterone inhibitors, calcium antagonists) were used by 8 smokers, 12 ex-smokers, and 22 never-smokers. Diuretics (thiazides, loop diuretics) were taken by 5 smokers, 4 ex-smokers, and 6 never-smokers. 100 mg aspirin daily was taken by 2 active smokers, 7 ex-smokers, and 7 never-smokers. Statins were taken by 5 active smokers, 10 ex-smokers, and 16 never-smokers.

Compared to controls, NSS scores were higher in ex-smokers (*p* = 0.026) and never-smokers (*p* = 0.036), but not in smokers (*p* = 0.233). There was no difference between the NSS scores of T2D patient groups. The pulse wave velocity (PWV), a marker of arterial stiffness and associated microangiopathy (Kim and Kim, 2019), was higher in ex-smokers compared to controls (*p* = 0.036).

### Serological Data

HbA1c levels were higher in never-smokers and ex-smokers when compared to controls (*p* = 0.003 and *p* = 0.005, respectively). There was no significant difference between HbA1c levels of T2D patient groups. High sensitivity Troponin T (hsTNT), a marker of cardiovascular disease, was higher in ex-smokers when compared to controls (*p* = 0.038). High-density lipoprotein (HDL) cholesterol was higher in smokers in comparison to ex-smokers (*p* = 0.016). No significant differences were found for cystatin c, glomerular filtration rate (GFR), N-terminal pro-brain natriuretic peptide (proBNP), total serum cholesterol, low-density lipoprotein (LDL) cholesterol, or triglycerides.

### Electrophysiological Data

Tibial NCV was higher in controls compared to ex-smokers and smokers (*p* = 0.047 and *p* = 0.018, respectively). Peroneal NCV was higher in controls compared to ex-smokers (*p* = 0.012) and to smokers (*p* = 0.033). No significant differences were found for sural NCV and amplitudes, tibial and peroneal CMAP, or tibial and peroneal DML. A detailed summary of all group comparisons on epidemiological, electrophysiological, and serological data is provided in Table 1.

**TABLE 1:** Comparison of imaging, clinical, electrophysiological, apparatus-bound, and serologic parameters between controls, type 2 diabetes (T2D) never-smokers, ex-smokers, and smokers.

Parameter	Controls	T2D never-smokers	T2D ex-smokers	T2D smokers	*P*-Value	*P*-Value controls vs. never-smokers	*P*-Value controls vs. ex-smokers	*P*-Value controls vs. smokers	*P*-Value never-smokers vs. ex-smokers	*P*-Value never-smokers vs. smokers	*P*-Value ex-smokers vs. smokers
Fractional anisotropy	0.52 ± 0.04	0.48 ± 0.06	0.43 ± 0.09	0.40 ± 0.08	<0.001	0.361	0.015	0.002	0.126	0.015	0.601
Radial diffusivity [10^–5^ mm^2^/s]	73.78 ± 11.94	77.71 ± 10.42	88.33 ± 24.44	93.53 ± 18.82	0.008	0.904	0.105	0.048	0.091	0.049	0.856
Axial diffusivity [10^–5^ mm^2^/s]	182.80 ± 23.58	171.80 ± 19.41	172.80 ± 30.86	174.40 ± 14.05	0.592	0.523	0.676	0.843	0.999	0.988	0.998
Women/Men	7w3m	18w22m	8w12m	5w5m	0.151	0.170	0.124	0.531	0.961	0.983	0.904
Age (years)	49.80 ± 12.89	57.44 ± 14.01	59.42 ± 6.87	53.20 ± 12.85	0.186	0.309	0.200	0.927	0.939	0.768	0.573
Body-mass index (kg/m^2^)	28.05 ± 5.69	28.45 ± 5.02	28.42 ± 5.52	30.48 ± 3.6	0.508	0.996	0.997	0.712	>0.999	0.677	0.730
NDS	0.30 ± 0.67	2.28 ± 2.85	2.94 ± 3.27	2.70 ± 2.67	0.103	0.22	0.097	0.22	0.777	0.892	0.892
NSS	0 ± 0	3.31 ± 3.21	3.78 ± 3.70	3.10 ± 3.41	0.027	0.036	0.026	0.232	>0.999	>0.999	>0.999
Tibial NCV (m/s)	47.10 ± 7.75	42.65 ± 5.07	40.76 ± 5.31	38.90 ± 8.01	0.017	0.151	0.047	0.018	0.488	0.227	0.489
Tibial CMAP (μV)	15.56 ± 8.18	12.91 ± 9.43	1212 ± 6.68	9.45 ± 5.91	0.427	0.808	0.728	0.361	0.988	0.648	0.851
Tibial DML (ms)	5.39 ± 3.18	7.46 ± 4.89	5.51 ± 3.63	6.53 ± 5.72	0.398	0.583	>0.999	0.944	0.465	0.940	0.943
Peroneal NCV (m/s)	46.00 ± 3.30	42.14 ± 6.17	39.24 ± 5.12	39.11 ± 6.60	0.008	0.247	0.012	0.033	0.525	0.852	>0.999
Peroneal CMAP (μV)	7.81 ± 2.08	5.83 ± 3.40	4.99 ± 2.79	4.54 ± 4.49	0.069	0.519	0.214	0.076	>0.999	0.900	>0.999
Peroneal DML (ms)	4.45 ± 1.81	6.43 ± 3.84	5.49 ± 2.87	6.67 ± 4.55	0.401	0.433	0.901	0.550	0.800	0.998	0.849.
Sural NCV (m/s)	48.78 ± 7.24	46.17 ± 6.00	45.92 ± 5.58	45.05 ± 9.94	0.693	>0.999	>0.999	>0.999	>0.999	>0.999	>0.999
Sural SNAP (μV)	9.78 ± 6.82	7.44 ± 4.49	5.16 ± 3.21	4.95 ± 3.48	0.054	0.515	0.077	0.134	0.348	0.501	>0.999
SAF (AU)	1.81 ± 0.37	2.15 ± 0.54	2.28 ± 0.48	2.32 ± 0.53	0.072	0.344	0.091	0.162	>0.999	>0.999	>0.999
Pulse wave velocity (m/s)	7.30 ± 1.56	8.26 ± 1.65	9.32 ± 1.78	8.75 ± 1.92	0.036	0.355	0.036	0.249	0.187	0.654	0.654
Intima-media thickness (ratio)	0.97 ± 0.21	0.92 ± 0.25	0.96 ± 0.23	0.75 ± 0.23	0.114	0.915	0.999	0.172	0.914	0.194	0.116
HbA1c (%)	5.45 ± 0.17	7.28 ± 1.90	7.05 ± 1.02	6.33 ± 1.29	0.002	0.003	0.005	0.467	>0.999	0.852	0.802
Cystatin C (mg/l)	0.73 ± 0.11	0.82 ± 0.17	0.9 ± 0.21	0.91 ± 0.26	0.185	0.568	0.173	0.177	0.551	0.543	0.996
Glomerular filtration rate (ml/min)	92.64 ± 8.97	88.39 ± 16.75	89.59 ± 17.90	79.36 ± 20.93	0.386	0.922	0.974	0.381	0.996	0.505	0.468
Total serum cholesterol (mg/dl)	204.80 ± 48.38	196.20 ± 41.63	189.60 ± 46.23	195.20 ± 45.71	0.865	0.958	0.854	0.968	0.956	>0.999	0.989
HDL cholesterol (mg/dl)	54.63 ± 12.06	55.15 ± 16.55	45.59 ± 10.64	67.80 ± 22.29	0.077	>0.999	0.614	>0.999	0.190	0.632	0.016
LDL cholesterol (mg/dl)	131.00 ± 39.60	110.90 ± 35.44	110.30 ± 39.37	107.60 ± 41.03	0.475	0.861	>0.999	0.879	>0.999	>0.999	>0.999
Triglycerides (mg/dl)	78.25 ± 15.35	79.95 ± 28.37	68.12 ± 14.69	77.90 ± 32.58	0.462	0.999	0.789	>0.999	0.412	0.998	0.770
Troponin T (pg/dl)	8.24 ± 8.69	7.93 ± 3.29	12.81 ± 7.62	10.90 ± 6.76	0.009	>0.999	0.038	0.530	0.066	>0.999	>0.999
proBNP (pg/dl)	24.13 ± 9.48	25.05 ± 9.08	27.00 ± 9.16	21.90 ± 8.48	0.554	0.994	0.881	0.955	0.882	0.762	0.496

*All values are displayed as mean ± standard deviation.*

*NDS, Neuropathy Disability Score; NSS, Neuropathy Symptom Score; NCV, Nerve Conduction Velocitie; CMAP, compound muscle action potential; DML, Peroneal Distal Motor Latencies; SNAP, Sensory Nerve Action Potential; SAF, Skin Auto Fluorescence; HbA1c, hemoglobin A1c; HDL, High-Density Lipoprotein; LDL, Low-Density Lipoprotein; proBNP, pro-Brain Natriuretic Peptide; m/s, meters per second; mm, millimeter, μV, microvolt; ms, milliseconds; AU, arbitrary units; mg/dl, milligram per deciliter; mg/l, milligram per liter; ml/min, milliliters per minute; pg/dl, picogram per deciliter.*

### Magnetic Resonance Neurography Imaging Data

#### Comparison of Groups and Correlation With Clinical Scores and Demographic Data

**FIGURE 3:**
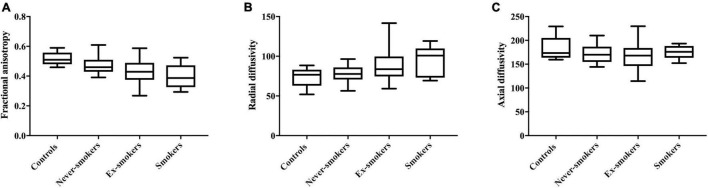
Parameters derived from diffusion weighted MRN in all groups. **(A)** Fractional anisotropy in controls (0.52 ± 0.04), never-smokers (0.48 ± 0.06), ex-smokers (0.43 ± 0.09), and smokers (0.40 ± 0.08); *F* = 6.324: *r*^2^ = 0.200; *p* < 0.001. **(B)** Radial diffusivity in controls (73.78 ± 11.94), never-smokers (77.71 ± 10.42), ex-smokers (88.33 ± 24.44), and smokers (93.53 ± 18.82); *F* = 4.264; *r*^2^ = 0.147; *p* = 0.008. **(C)** Axial diffusivity in controls (182.80 ± 23.58), never-smokers (171.80 ± 19.41), ex-smokers (172.80 ± 30.86), and smokers (174.40 ± 14.05); *F* = 1.845; *r*^2^ = 0.026; *p* = 0.570.

FA as the main imaging parameter for structural nerve integrity, was higher in controls when compared to ex-smokers (*p* = 0.015) and smokers (*p* = 0.002) but not when compared to never-smokers (*p* = 0.361; Figure 3A). FA was also higher in never-smokers when compared to smokers (*p* = 0.015). A summary of all group comparisons for imaging data is provided in Table 1. In controls, FA showed negative correlations with age (*r* = −0.75; *p* = 0.013), likewise in never smokers (*r* = −0.50; *p* = 0.001). This finding could not be reproduced for ex-smokers and smokers. In smokers with T2D, FA showed negative correlations with BMI (*r* = −0.75; *p* = 0.013). In never-smokers, ex-smokers and smokers with T2D, FA was negatively correlated with the NDS score (*r* = −0.49; *p* = 0.001, *r* = −0.52; *p* = 0.031, and *r* = −0.70; *p* = 0.024, respectively). In smokers, FA also showed a negative correlation with the NSS score (*r* = −0.69, *p* = 0.028). No correlations were found with pack years or the average number of cigarettes per day in smokers. A detailed survey of correlations between FA and all other parameters acquired is provided in Table 2.

**TABLE 2:** Correlation of the sciatic nerve’s fractional anisotropy (FA) with demographic, clinical, apparatus-bound, and serological parameters.

	FA controls		FA T2D never-smokers		FA T2D ex-smokers		FA T2D smokers	
	*r*	*p*	*r*	*p*	*r*	*p*	*r*	*p*
Radial diffusivity	–0.26	0.469	–0.67	<0.001	–0.89	<0.001	–0.80	0.010
Axial diffusivity	0.36	0.305	0.29	0.071	–0.35	0.144	–0.65	0.044
Sex	0.01	0.974	0.16	0.328	0.22	0.347	0.29	0.410
Age (years)	–0.75	0.013	–0.50	0.001	–0.21	0.372	–0.47	0.173
Body-mass index (kg/m^2^)	0.17	0.635	–0.29	0.074	–0.003	0.990	–0.75	0.013
NDS	–0.02	0.955	–0.50	0.001	–0.523	0.031	–0.70	0.024
NSS	*n.a*.	n.a.	–0.12	0.479	–0.06	0.812	–0.69	0.028
Tibial NCV (m/s)	0.41	0.236	0.34	0.042	0.60	0.007	0.67	0.034
Tibial CMAP (μV)	0.09	0.806	0.45	0.006	0.55	0.014	0.51	0.136
Tibial DML (ms)	–0.21	0.553	0.06	0.710	–0.32	0.180	–0.59	0.074
Peroneal NCV (m/s)	0.53	0.117	0.41	0.014	0.70	0.001	0.85	0.003
Peroneal CMAP (μV)	0.15	0.675	0.58	<0.001	0.64	0.003	0.64	0.066
Peroneal DML (ms)	–0.12	0.733	0.01	0.938	–0.42	0.075	–0.58	0.099
Sural NCV (m/s)	–0.06	0.877	–0.03	0.887	–0.15	0.597	–0.05	0.927
Sural SNAP (μV)	0.38	0.279	0.32	0.069	0.34	0.167	0.26	0.540
SAF (AU)	–0.41	0.241	–0.39	0.020	–0.07	0.765	–0.84	0.004
PWV (m/s)	–0.70	0.037	–0.45	0.004	–0.51	0.027	–0.59	0.094
IMT	–0.61	0.061	–0.09	0.599	–0.26	0.289	–0.69	0.039
HbA1c (%)	–0.47	0.242	0.13	0.422	0.05	0.859	–0.35	0.329
Cystatin C (mg/l)	0.41	0.317	–0.37	0.038	0.07	0.800	0.31	0.420
Glomerular filtration rate (ml/min)	–0.37	0.369	0.45	0.012	–0.17	0.512	–0.26	0.506
Total serum cholesterol (mg/dl)	–0.12	0.770	0.26	0.109	–0.20	0.423	–0.30	0.397
HDL cholesterol (mg/dl)	–0.29	0.489	0.08	0.622	–0.30	0.229	–0.34	0.343
LDL cholesterol (mg/dl)	–0.03	0.937	0.24	0.149	–0.11	0.687	–0.24	0.502
Triglycerides (mg/dl)	–0.13	0.759	0.27	0.093	–0.08	0.745	0.46	0.178
Troponin T (pg/dl)	–0.43	0.292	–0.26	0.153	0.20	0.432	–0.36	0.341
proBNP (pg/dl)	–0.15	0.716	–0.25	0.169	0.23	0.368	0.38	0.309
Years since smoke stop	*n.a*.	n.a.	*n.a*.	n.a.	–0.11	0.672	*n.a*.	n.a.
Cigarettes/day	*n.a*.	n.a.	*n.a*.	n.a.	*n.a*.	n.a.	–0.23	0.520
Years smoking	*n.a*.	n.a.	*n.a*.	n.a.	*n.a*.	n.a.	–0.37	0.365
Pack years	*n.a*.	n.a.	*n.a*.	n.a.	*n.a*.	n.a.	–0.32	0.445

*NDS, Neuropathy Disability Score; NSS, Neuropathy Symptom Score; NCV, Nerve Conduction Velocitie; CMAP, compound muscle action potential; DML, Peroneal Distal Motor Latencies; SNAP, Sensory Nerve Action Potential; SAF, Skin Auto Fluorescence; PWV, Pulse Wave Velocity; IMT, Intima Media Thickness; HbA1c, hemoglobin A1c; HDL, High-Density Lipoprotein; LDL, Low-Density Lipoprotein; proBNP, pro-Brain Natriuretic Peptide; m/s, meters per second; μV, microvolt; ms, milliseconds; AU, arbitrary units; mg/dl, milligram per deciliter; mg/l, milligram per liter; ml/min, milliliters per minute; pg/dl, picogram per deciliter.*

For RD, a parameter indicative for the damage to myelin, ANOVA showed higher values in smokers compared to controls (*p* = 0.048) and never-smokers (*p* = 0.049; Figure 3B).

In never-smokers, RD was correlated with age (*r* = 0.44; *p* = 0.005). In smokers, RD was correlated with the BMI (*r* = 0.68; *p* = 0.042). RD was positively correlated with the NDS score in never-smokers (*r* = 0.39; *p* = 0.017) and smokers (*r* = 0.68; *p* = 0.046), but not in ex-smokers (*r* = 0.275; *p* = 0.286). No correlations were found with the NSS score. A detailed survey of correlations between RD and all other parameters is provided in Table 3.

**TABLE 3:** Correlation of the sciatic nerve’s radial diffusivity (RD; in 10^–5^ mm^2^/s) with demographic, clinical, apparatus-bound, and serological parameters.

	RD controls		RD T2D never-smokers		RD T2D ex-smokers		RD T2D smokers	
	*r*	*p*	*r*	*p*	*r*	*p*	*r*	*p*
Fractional anisotropy	–0.26	0.469	–0.67	<0.001	–0.89	<0.001	–0.80	0.010
Axial diffusivity	0.70	0.023	0.47	0.003	0.72	0.001	0.86	0.003
Sex	0.11	0.760	–0.07	0.655	–0.24	0.307	–0.17	0.655
Age (years)	–0.04	0.916	0.44	0.005	0.17	0.467	0.22	0.571
Body-mass index (kg/m^2^)	–0.27	0.456	0.06	0.732	–0.09	0.696	0.68	0.042
NDS	–0.43	0.221	0.39	0.017	0.28	0.286	0.67	0.046
NSS			0.26	0.114	–0.22	0.403	0.44	0.242
Tibial NCV (m/s)	–0.33	0.358	–0.26	0.125	–0.50	0.028	–0.48	0.186
Tibial CMAP (μV)	<0.01	0.989	–0.37	0.031	–0.59	0.008	–0.78	0.013
Tibial DML (ms)	0.12	0.749	–0.21	0.233	0.42	0.076	0.72	0.028
Peroneal NCV (m/s)	0.14	0.696	–0.34	0.046	–0.49	0.034	–0.86	0.006
Peroneal CMAP (μV)	0.27	0.456	–0.51	0.002	–0.56	0.012	–0.83	0.012
Peroneal DML (ms)	0.38	0.282	–0.09	0.625	0.38	0.112	0.76	0.029
Sural NCV (m/s)	0.35	0.322	0.10	0.612	0.31	0.266	–0.26	0.670
Sural SNAP (μV)	0.04	0.908	–0.26	0.153	–0.07	0.770	0.57	0.182
SAF (AU)	–0.19	0.591	0.44	0.009	0.07	0.765	0.59	0.112
PWV (m/s)	–0.15	0.701	0.19	0.269	0.33	0.171	0.56	0.152
IMT (ratio)	0.02	0.957	0.28	0.084	0.25	0.317	0.74	0.035
HbA1c (%)	–0.36	0.383	–0.30	0.060	–0.09	0.720	0.68	0.046
Cystatin C (mg/l)	0.04	0.920	0.11	0.566	–0.15	0.571	0.04	0.932
Glomerular filtration rate (ml/min)	<0.01	0.994	–0.13	0.499	0.21	0.428	–0.01	0.988
Total serum cholesterol (mg/dl)	0.36	0.384	–0.29	0.073	–0.06	0.802	0.63	0.068
HDL cholesterol (mg/dl)	–0.11	0.792	–0.05	0.757	0.14	0.593	0.43	0.243
LDL cholesterol (mg/dl)	0.42	0.295	–0.21	0.216	–0.11	0.666	0.44	0.234
Triglycerides (mg/dl)	0.18	0.670	–0.33	0.043	–0.01	0.964	0.20	0.608
Troponin T (pg/dl)	–0.55	0.155	0.30	0.108	–0.29	0.241	0.71	0.040
proBNP (pg/dl)	0.06	0.879	0.26	0.166	–0.27	0.276	–0.46	0.257
Years since smoke stop	*n.a*.	n.a.	*n.a*.	n.a.	0.03	0.902	*n.a*.	n.a.
Cigarettes/day	*n.a*.	n.a.	*n.a*.	n.a.	*n.a*.	n.a.	0.31	0.422
Years smoking	*n.a*.	n.a.	*n.a*.	n.a.	*n.a*.	n.a.	–0.21	0.651
Pack years	*n.a*.	n.a.	*n.a*.	n.a.	*n.a*.	n.a.	0.03	0.946

*NDS, Neuropathy Disability Score; NSS, Neuropathy Symptom Score; NCV, Nerve Conduction Velocitie; CMAP, compound muscle action potential; DML, Peroneal Distal Motor Latencies; SNAP, Sensory Nerve Action Potential; SAF, Skin Auto Fluorescence; PWV, Pulse Wave Velocity; IMT, Intima Media Thickness; HbA1c, hemoglobin A1c; HDL, High-Density Lipoprotein; LDL, Low-Density Lipoprotein; proBNP, pro-Brain Natriuretic Peptide; m/s, meters per second; μV, microvolt; mm, millimeter; ms, milliseconds; AU, arbitrary units; mg/dl, milligram per deciliter; mg/l, milligram per liter; ml/min, milliliters per minute; pg/dl, picogram per deciliter.*

For all groups, no significant differences were found for AD, a parameter supposed to represent axonal integrity (Figure 3C). In smokers, AD was correlated with the BMI (*r* = 0.76; *p* = 0.011). No other correlations were found for AD with any of the acquired clinical scores or demographic data. A detailed survey of correlations of AD and with all other parameters is provided in Table 4.

**TABLE 4:** Correlation of the sciatic nerve’s axial diffusivity (AD; in 10**^–^**^5^ mm^2^/s) with demographic, clinical, apparatus-bound, and serological parameters.

	AD controls		AD T2D never-smokers		AD T2D ex-smokers		AD T2D smokers	
	*r*	*p*	*r*	*p*	*r*	*p*	*r*	*p*
Fractional anisotropy	0.36	0.305	0.29	0.071	–0.35	0.144	–0.65	0.044
Radial diffusivity	0.70	0.023	0.47	0.003	0.72	0.001	0.86	0.003
Sex	0.36	0.306	0.02	0.918	–0.07	0.767	0.13	0.727
Age (years)	–0.48	0.166	–0.01	0.936	–0.01	0.965	0.13	0.721
Body-mass index (kg/m^2^)	–0.45	0.193	–0.32	0.048	–0.23	0.342	0.76	0.011
NDS	–0.42	0.225	–0.05	0.779	–0.18	0.493	0.44	0.207
NSS			0.26	0.106	–0.48	0.049	0.44	0.199
Tibial NCV (m/s)	–0.09	0.806	0.02	0.906	–0.17	0.511	–0.26	0.466
Tibial CMAP (μV)	0.04	0.916	0.09	0.595	–0.41	0.088	–0.61	0.062
Tibial DML (ms)	0.11	0.760	–0.18	0.305	0.51	0.032	0.64	0.046
Peroneal NCV (m/s)	0.36	0.313	0.04	0.808	0.08	0.753	–0.73	0.027
Peroneal CMAP (μV)	0.42	0.225	0.06	0.736	–0.07	0.776	–0.85	0.004
Peroneal DML (ms)	0.32	0.366	–0.10	0.573	0.08	0.763	0.66	0.053
Sural NCV (m/s)	0.54	0.110	0.02	0.939	0.29	0.311	0.17	0.750
Sural SNAP (μV)	0.56	0.090	0.08	0.676	0.39	0.120	0.09	0.834
SAF (AU)	–0.55	0.101	0.22	0.205	–0.07	0.786	0.57	0.109
PWV (m/s)	–0.45	0.225	–0.19	0.264	<0.01	0.985	0.30	0.438
IMT	–0.22	0.548	0.21	0.201	–0.04	0.882	0.62	0.077
HbA1c (%)	–0.22	0.605	–0.28	0.076	–0.13	0.630	0.69	0.027
Cystatin C (mg/l)	0.44	0.271	–0.31	0.086	–0.19	0.485	–0.05	0.900
Glomerular filtration rate (ml/min)	–0.38	0.355	0.38	0.040	0.20	0.466	0.21	0.594
Total serum cholesterol (mg/dl)	0.38	0.355	–0.02	0.920	–0.30	0.246	0.48	0.157
HDL cholesterol (mg/dl)	0.03	0.938	–0.03	0.872	–0.06	0.830	0.10	0.789
LDL cholesterol (mg/dl)	0.38	0.359	0.14	0.417	–0.28	0.289	0.44	0.201
Triglycerides (mg/dl)	0.30	0.468	–0.11	0.518	–0.12	0.658	0.24	0.512
Troponin T (pg/dl)	–0.44	0.271	0.06	0.769	–0.34	0.180	0.42	0.255
proBNP (pg/dl)	–0.01	0.976	0.01	0.974	–0.30	0.248	–0.58	0.105
Years since smoke stop	*n.a*.	n.a.	*n.a*.	n.a.	–0.14	0.590	*n.a*.	n.a.
Cigarettes/day	*n.a*.	n.a.	*n.a*.	n.a.	*n.a*.	n.a.	0.41	0.245
Years smoking	*n.a*.	n.a.	*n.a*.	n.a.	*n.a*.	n.a.	–0.22	0.600
Pack years	*n.a*.	n.a.	*n.a*.	n.a.	*n.a*.	n.a.	–0.05	0.914

*NDS, Neuropathy Disability Score; NSS, Neuropathy Symptom Score; NCV, Nerve Conduction Velocitie; CMAP, compound muscle action potential; DML, Peroneal Distal Motor Latencies; SNAP, Sensory Nerve Action Potential; SAF, Skin Auto Fluorescence; PWV, Pulse Wave Velocity; IMT, Intima Media Thickness; HbA1c, hemoglobin A1c; HDL, High-Density Lipoprotein; LDL, Low-Density Lipoprotein; proBNP, pro-Brain Natriuretic Peptide; m/s, meters per second; μV, microvolt; mm, millimeter; ms, milliseconds; AU, arbitrary units; mg/l, milligram per liter; ml/min, milliliters per minute; mg/dl, milligram per deciliter; pg/dl, picogram per deciliter.*

#### Correlation With Electrophysiological Data

FA showed positive correlations with tibial and peroneal NCV in never-smokers (*r* = 0.34; *p* = 0.042 and *r* = 0.41; *p* = 0.014, respectively), ex-smokers (*r* = 0.60; *p* = 0.007, and *r* = 0.70; *p* < 0.001, respectively), and smokers (*r* = 0.67; *p* = 0.034 and *r* = 0.85; *p* = 0.003, respectively) with T2D. Further positive correlations were found between FA and tibial and peroneal CMAP in never-smokers (*r* = 0.45; *p* = 0.006 and *r* = 0.58; *p* < 0.001, respectively) and ex-smokers (*r* = 0.55; *p* = 0.014 and *r* = 0.64; *p* = 0.003, respectively), while a similar trend was found in smokers (*r* = 0.51 *p* = 0.136 and *r* = 0.64; *p* = 0.066, respectively).

RD showed correlations with peroneal NCV and CMAP in never-smokers (*r* = −0.34; *p* = 0.046 and *r* = −0.51; *p* = 0.002), ex-smokers (*r* = −0.49; *p* = 0.034 and *r* = −0.56; *p* = 0.012) and smokers (*r* = −0.86; *p* = 0.033 and *r* = −0.83; *p* = 0.012) as well as correlations with tibial CMAP in never smokers (*r* = −0.37; *p* = 0.031), ex-smokers (*r* = −0.59; *p* = 0.008) and smokers (*r* = −0.78; *p* = 0.013) with T2D.

AD was correlated with tibial DML in ex-smokers and smokers (*r* = 0.51; *p* = 0.032 and *r* = 0.64; *p* = 0.046, respectively) with T2D. In smokers with T2D, AD was further correlated with peroneal NCV (*r* = −0.73; *p* = 0.027) and peroneal CMAP (*r* = −0.85; *p* = 0.004).

#### Correlation With Additional Paraclinical Data

FA showed negative correlations with the pulse wave velocity in controls (*r* = −0.70; *p* = 0.037), never-smokers (*r* = −0.45; *p* = 0.004), ex-smokers (*r* = −0.51; *p* = 0.027), and a similar trend in smokers (*r* = −0.59; *p* = 0.094) with T2D. In smokers with T2D, IMT as a surrogate marker of cardiovascular disease in diabetes (Sibal et al., 2011) was negatively correlated with FA (*r* = −0.69; *p* = 0.039). Negative correlations were found between FA and SAF, a measure of the tissue deposition of advanced glycation end product and therefore of the degree of glycemic stress in diabetes, in never-smokers (*r* = −0.39; *p* = 0.020) and smokers (*r* = −0.84; *p* = 0.004), but not in ex-smokers with T2D (*r* = −0.07; *p* = 0.765).

RD was positively correlated with the IMT in smokers (*r* = 0.74; *p* = 0.035). No correlations were found between RD and PWV. RD was positively correlated with skin auto-fluorescence in never-smokers (*r* = 0.44; *p* = 0.009) and a similar trend was seen in smokers (*r* = 0.59; *p* = 0.122) but not in ex-smokers (*r* = 0.07; *p* = 0.765).

#### Correlation With Serologic Data

In never-smokers, FA showed negative correlations with cystatin c and positive correlations with GFR (*r* = −0.37; *p* = 0.038 and *r* = 0.45; *p* = 0.012). No correlations were found between FA and HbA1c levels, cholesterol levels or triglycerides.

In smokers, RD was correlated with HbA1c levels (*r* = 0.68; *p* = 0.046) and hsTNT (*r* = 0.71; *p* = 0.048). No further correlations of AD with serologic parameters were found in any of the groups. A detailed summary of all correlations of imaging parameters is given in Tables 2–4.

## Discussion

The key findings of this study were that (i) the sciatic nerve’s FA is lower in smokers and ex-smokers with T2D compared to age-matched controls, and that the FA in smokers with T2D is lower than in never-smokers with T2D; (ii) in all T2D patient groups, FA was negatively correlated with the NDS, tibial and peroneal NCV and CMAP, and PWV; (iii) in never-smokers and smokers, FA was negatively correlated with SAF, whereas in ex-smokers no such correlation could be found; and (iv) in smokers, RD was positively correlated with hsTNT, SAF, BMI, and HbA1c levels.

The finding that the sciatic nerve’s FA was lower in smokers compared to controls and never-smokers suggests that cigarette smoking contributes to nerve damage in patients with T2D, and that, therefore, to quit smoking may pose a useful preventive measure to slow the progression of DPN, as suggested by previous studies (Kar et al., 2016). The finding of a lower FA in ex-smokers compared to controls suggests that the negative effect of smoking on structural nerve integrity in T2D is not completely reversible once a patient quits smoking, supporting previous studies that found smoking prevention in adolescents with diabetes to be of importance with regards to the development and progression of DPN (Jaiswal et al., 2017; Christensen et al., 2020). The finding that RD, but not AD was higher in smokers with T2D when compared to controls and never-smokers with T2D, suggests that smoking primarily causes damage to the myelin sheath in T2D, since RD has previously been shown to be a marker for myelin integrity (Heckel et al., 2015; Vaeggemose et al., 2017; Kurz et al., 2018). The correlations of FA and RD with electrophysiological parameters and clinical scores indicate that both parameters pose reliable parameters for structural nerve integrity in T2D as suggested by recent studies (Jende et al., 2021).

The finding that FA was negatively correlated with SAF in smokers, but not in ex-smokers, suggests that the deposition of advanced glycation end products in or around peripheral nerves and subsequent myelin damage may be more relevant in active smokers than in ex-smokers. In contrast, the absence of a correlation between FA and SAF in ex-smokers and the findings that both hsTNT and PWV, markers of microangiopathy in T2D (Jende et al., 2020a), are higher in ex-smokers versus controls, while PWV was not significantly associated with FA decrease in smokers, may suggest that microvascular impairment associated nerve damage predominates over effects of hyperglycemia in T2D ex-smokers.

The correlation of RD with IMT, hsTNT and HbA1c levels in smokers further supports the hypothesis that hyperglycemia, macroangiopathy, and microangiopathy induced by smoking contribute to myelin damage in T2D in the sense of a “triple hit” (Nilsson et al., 2004; Christensen et al., 2020). The finding that the sciatic nerve’s RD was positively correlated with the BMI in smokers further supports the hypothesis that the impact of smoking on nerve integrity is worse in obese patients with T2D (Jaiswal et al., 2017).

The results of this study are of importance with regard to the effect of glucose control on the progression of DPN in T2D: since former studies have found that normalization of blood glucose levels in T2D is not beneficial for slowing the progression of DPN, it might be of interest to future studies to assess whether the effect of glucose control in T2D differs between smokers, ex-smokers and never-smokers.

This explorative study is limited by the fact that only cross-sectional data were analyzed, which does not allow for causative conclusions on the effects of smoking on myelin damage in T2D. Another limitation is the fact that our sample size, in particular the number of active smokers, is too small for multivariate analysis of all potential confounders. One must consider, however, that all patient groups were matched for gender, age, BMI, HbA1c levels, parameters of renal function, and total serum cholesterol. It is therefore unlikely that the effects found in our cohort are caused by other effects than those induced by cigarette smoking.

One may of course argue that we did not separate DPN patients from patients without DPN. However, it should be noted that all patient groups were matched for NDS and that previous MRN imaging studies have found that DPN is a continuous process during which nerve lesions accumulate in asymptomatic patients who start to experience symptoms once a certain amount of structural nerve damage is reached (Groener et al., 2019). Therefore, assessing the sciatic nerve’s structural integrity with MRN imaging parameters allows for more accurate correlations and group comparisons than dividing patients into two groups based on clinical scores (Vaeggemose et al., 2017; Jende et al., 2020a).

In summary, this study is the first to find that structural nerve integrity assessed by MR neurography is lower in smokers with T2D compared to ex-smokers with T2D and age-matched controls, which is most likely due to damage to the affected nerves’ myelin sheath. Our findings suggest that in smokers with T2D, hyperglycemia, obesity, micro- and macroangiopathy contribute to myelin damage, whereas in ex-smokers microangiopathy appears to be the main contributing factor. Our results indicate that cigarette smoking is harmful to peripheral nerves in T2D and that, therefore, interventions to stop smoking may be promising preventive measures to slow down the progression of DPN. Further longitudinal studies are required to verify this hypothesis.

## Data Availability Statement

The datasets presented in this article are not readily available because they contain sensitive patient information. The data supporting the conclusions of this article will be made available upon reasonable request by any qualified researcher. Requests to access the datasets should be directed to FK, felix.kurz@med.uni-heidelberg.de.

## Ethics Statement

The studies involving human participants were reviewed and approved by Ethikkommission der medizinischen Fakultät der Universität Heidelberg, Alte Glockengießerei 11/1, 69115 Heidelberg, Germany. The patients/participants provided their written informed consent to participate in this study.

## Author Contributions

JJ, MB, SH, PN, and FK designed and coordinated the study. JJ, CM, JG, AJ, and FK contributed to the organization of participants. JJ, AJ, and FK collected MR data. FK developed image analysis tools. ZK, JG, and SK collected clinical, serological, and electrophysiological data. JJ and FK analyzed the data and wrote the manuscript with input from all coauthors. All authors contributed to the article and approved the submitted version.

## Conflict of Interest

The authors declare that the research was conducted in the absence of any commercial or financial relationships that could be construed as a potential conflict of interest.

## Publisher’s Note

All claims expressed in this article are solely those of the authors and do not necessarily represent those of their affiliated organizations, or those of the publisher, the editors and the reviewers. Any product that may be evaluated in this article, or claim that may be made by its manufacturer, is not guaranteed or endorsed by the publisher.

## Abbreviations

DPN: diabetic polyneuropathy
T2D: type 2 diabetes
FA: fractional anisotropy
MRN: magnetic resonance neurography
NDS: Neuropathy Disability Score
NSS: Neuropathy Symptom Score.
